# Prevention of lymphoceles using peritoneal flaps during robotic‐assisted radical prostatectomy with pelvic lymph node dissection: A systematic review and meta‐analysis

**DOI:** 10.1002/bco2.70126

**Published:** 2026-02-26

**Authors:** Huseyin Yildiz, Mohammed Zain Ulabedin Adhoni, Kevin Byrnes, Benjamin Lamb, David I. Lee, Mohammed Shahait

**Affiliations:** ^1^ Department of Urology Darent Valley Hospital Dartford UK; ^2^ Department of Urology The Royal London Hospital London UK; ^3^ Centre for Cancer Cell and Molecular Biology, Barts Cancer Institute Queen Mary University of London London UK; ^4^ Department of Urology University of California, Irvine Irvine CA USA

**Keywords:** lymphocele, pelvic lymph node dissection, peritoneal flap, prostate cancer, robotic‐assisted radical prostatectomy, systematic review and meta‐analysis

## Abstract

**Objective:**

The study aims to assess whether the use of a peritoneal flap (PF) during robotic‐assisted radical prostatectomy (RARP) with pelvic lymph node dissection (PLND) reduces the incidence of lymphoceles compared to the standard surgical approach without a flap.

**Methods:**

The review was prospectively registered on PROSPERO (CRD420251052120). A systematic search of PubMed, MEDLINE, Embase, Scopus, Web of Science, CENTRAL and Google Scholar was performed up to May 2025. Eligible studies were randomised controlled trials (RCTs) or observational studies comparing PF use with the standard surgical technique without the flap during RARP with PLND. Primary outcomes were symptomatic, asymptomatic, total lymphoceles and lymphoceles requiring intervention. Secondary outcomes included complications, operative time, blood loss, positive surgical margins and hospital stay.

**Results:**

Fourteen studies (six RCTs, eight observational) including 7316 patients were analysed, with 2997 receiving the PF and 4319 receiving the standard technique. PF use was associated with a significantly lower incidence of symptomatic, asymptomatic, total lymphoceles and lymphoceles requiring intervention. PF use reduces overall complications without significantly increasing operative time, hospital stay or positive margins. Intraoperative blood loss was slightly lower in the standard group.

**Conclusions:**

PF use during RARP with PLND significantly reduces the incidence of lymphoceles and postoperative complications without compromising oncological or perioperative outcomes. These findings support PF use as a safe and effective technique for preventing lymphoceles.

## 1 INTRODUCTION

The European Association of Urology (EAU) and the American Urology Association (AUA) recommend radical prostatectomy (RP) with extended pelvic lymph node dissection (ePLND) in patients with intermediate risk or high‐risk locally advanced prostate cancer.^1^, ^2^ Robotic‐assisted radical prostatectomy (RARP) with PLND is the preferred surgical approach for these patients. However, PLND is associated with surgical complications, with lymphoceles being the most common.^3^ Lymphoceles commonly occur as a result of lymphatic fluid leakage from disrupted lymphatic vessels.^4^ Lymphoceles are not absorbed as they are not in communication with epithelial cells.^4^ Lymphoceles are reported in 9% to 51% of patients following RARP with PLDN.^5^, ^6^ Although most lymphoceles are asymptomatic, 2%–15% of patients with lymphoceles present with symptoms.^5^, ^6^ Symptoms include lower limb oedema, pain, constipation, urinary retention, infection, deep vein thrombosis (DVT) and pulmonary embolism (PE).^6^, ^7^ Symptomatic lymphoceles may require drainage through invasive procedures such as percutaneous drainage or surgical fenestration.^7^

In 2015, Lebeis et al. developed the peritoneal interposition flap (PIF) surgical technique, which demonstrated positive results in reducing lymphocele formation following RARP with PLND.^8^ The PIF manipulates the peritoneum to facilitate the flow of lymphatic fluid from the PLND bed and into the peritoneal cavity, where it is absorbed by the peritoneal membrane. Since then, there have been various studies and variations of surgical techniques that similarly allow for the continuous flow of accumulated lymphatic fluid into the peritoneal cavity for absorption. Multiple systematic reviews and meta‐analyses investigating the effectiveness of the peritoneal flap (PF) in preventing lymphoceles have been published with promising results.^9^, ^10^, ^11^, ^12^, ^13^ However, due to the publication of new high‐volume studies and variability in reported outcomes, an updated synthesis of the current evidence is required. Therefore, the aim of this systematic review and meta‐analysis is to assess the safety of the PF and to determine whether its use during RARP and PLND reduces lymphocele formation compared to the standard surgical approach without the PF.

## 2 METHODS

The protocol for this systematic review and meta‐analysis was prepared in advance and registered with the International Prospective Register of Systematic Reviews (PROSPERO; Registration ID: CRD420251052120). The review was conducted in accordance with the methodological guidance outlined in the Cochrane Handbook for Systematic Reviews of Interventions. Reporting was carried out in line with PRISMA (Preferred Reporting Items for Systematic Reviews and Meta‐Analysis) 2020 guidelines.

### 2.1 Search strategy

The following electronic databases were searched: PubMed, MEDLINE (via Ovid), Embase, CENTRAL (Cochrane Central Register of Controlled Trials), Scopus, Web of Science and Google Scholar. Database searches were carried out from their date of inception up until 20th May 2025. The search strategy incorporated both free‐text keywords and controlled vocabulary terms (e.g., MeSH), covering three core areas: lymphocele, RARP and PF techniques. Boolean operators and truncation symbols were employed to enhance search sensitivity and to account for variations in terminology. Database‐specific adaptions were made to ensure compatibility with each platform's indexing system. Studies were limited to those involving human participants and published in English. Editorials and conference abstracts were excluded. A full breakdown of the search strings and terms used for each database can be found within the supplementary material Data (S1).

### 2.2 Eligibility criteria

All studies were screened independently by two reviewers, with discrepancies resolved through discussion and consensus. Full‐text articles were retrieved for studies that met the inclusion criteria or where relevance could not be determined from the title and abstract alone. The relevance of each study was determined based on the inclusion and exclusion criteria, designed using the PICOS model (Patient Intervention Comparison Outcome Study). Studies that did not meet the inclusion criteria or studies that met the exclusion criteria were excluded.

### 2.3 Data extraction

Data extraction was carried out independently by two reviewers using a Microsoft Excel spreadsheet. The following data were collected in this study: study characteristics (author, year, study design), baseline participant demographics (mean age, BMI), surgical technique employed and sample size. Primary outcome measures extracted included the incidence of symptomatic lymphoceles, asymptomatic lymphoceles, all lymphoceles and lymphoceles requiring intervention. Secondary outcomes extracted included operative time, hospital stay duration, blood loss, positive surgical margin rates and complication rates. Complications were further stratified according to the Clavien‐Dindo classification, including total complications and those classified as above and including grade three (major complications) or below grade three (minor complications). In addition, the duration of follow‐up for each study was recorded. Follow‐up durations reported in days were converted to months using a standardised conversion. Studies providing data in median and ranges were converted to mean and standard deviation using Wan's method.

### 2.4 Quality assessment

The risk of bias for each included study was independently assessed by two reviewers using established critical appraisal protocols. Cochrane risk of bias tool version 2 (RoB 2.0) was used for randomised controlled trials (RCTs) (Table 1). Newcastle–Ottawa Scale was used to evaluate the quality of the observational studies (Table 2). Any disagreements between reviewers were resolved through discussion until consensus was reached.

**TABLE 1:** RoB 2 assessment of the included RCTs.

Study ID	D1	D2	D3	D4	D5	Overall
Pose et al.^14^	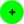	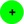	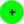	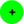	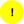	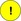
Neuberger et al.^15^	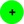	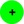	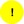	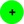	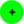	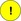
Gloger et al.^16^	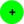	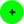	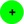	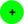	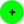	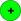
Student Jr et al.^17^	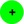	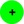	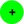	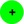	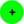	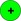
Wagner et al.^18^	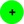	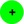	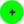	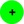	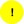	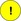
Brundl et al.^19^	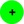	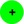	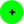	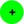	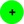	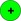

*Note*: 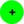 = low risk; 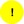 = some concerns; 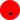 = high risk; D1 = randomisation process; D2 = deviations from the intended interventions; D3 = missing outcome data; D4 = measurement of the outcome; D5 = selection of the reported result.

**TABLE 2:** Newcastle–Ottawa scale for cohort studies.

Study	Representativeness of the exposed cohort	Selection of the nonexposed cohort	Ascertainment of exposure	Demonstration that the outcome of interest was not present at the start of the study	Comparability of cohorts on the basis of the design or analysis		Assessment of outcome	Was follow‐up long enough for outcomes to occur	Adequacy of follow up of cohorts	Total score (out of 9)
Harland et al.^20^	1	1	1	0	1	0	1	1	1	7
Mathew et al.^21^	1	1	1	0	1	1	1	1	1	8
Lee et al.^22^	1	1	1	0	1	0	1	1	1	7
Stolzenburg et al.^23^	1	1	1	0	1	0	1	1	1	7
Moro et al.^24^	1	1	1	0	1	0	1	1	1	7
Lebeis et al.^8^	1	1	1	0	1	1	1	1	1	8
Gamal et al.^25^	1	1	1	0	1	0	1	1	1	7
Gozen et al.^26^	1	1	1	0	1	1	1	1	1	8

### 2.5 Certainty of evidence

The certainty of evidence for symptomatic, asymptomatic and total lymphoceles was separately assessed for both the included RCTs and the observational studies using the GRADE (Grading of Recommendations, Assessment, Development, and Evaluation) framework. Evidence from RCTs was initially rated as high certainty, while that from observational studies was rated as low. Each outcome was then evaluated across five domains: risk of bias, inconsistency, indirectness, imprecision and publication bias. Where concerns were identified, the certainty rating was downgraded accordingly. A summary of GRADE ratings for all outcomes is provided in Table 3.

**TABLE 3:** Grade assessment of symptomatic lymphoceles, asymptomatic lymphoceles and total lymphoceles for the included RCTs and observational studies.

Outcomes	№ of participants (studies) Follow‐up	Certainty of the evidence (GRADE)	Relative effect (95% CI)	Anticipated absolute effects	
				Risk with no peritoneal flap	Risk difference with peritoneal flap
Symptomatic lymphoceles (RCTs)	2764 (6 RCTs)	⨁⨁⨁◯ Moderate^a^	OR 0.54 (0.35 to 0.85)	183 per 1000	75 fewer per 1000 (110 fewer to 23 fewer)
Asymptomatic lymphoceles (RCTs)	1712 (5 RCTs)	⨁⨁⨁◯ Moderate^b^ ^,^ ^c^	OR 0.52 (0.38 to 0.73)	200 per 1000	85 fewer per 1000 (113 fewer to 46 fewer)
Total lymphoceles (RCTs)	2764 (6 RCTs)	⨁⨁⨁◯ Moderate^a^	OR 0.52 (0.37 to 0.72)	306 per 1000	120 fewer per 1000 (166 fewer to 65 fewer)
Symptomatic lymphoceles (Observational studies)	3720 (5 non‐randomised studies)	⨁⨁◯◯ Low^d^ ^,^ ^e^	OR 0.18 (0.08 to 0.37)	34 per 1000	28 fewer per 1000 (31 fewer to 21 fewer)
Asymptomatic lymphoceles (Observational studies)	3349 (4 non‐randomised studies)	⨁◯◯◯ Very low^c^ ^,^ ^d^ ^,^ ^f^	OR 0.59 (0.26 to 1.32)	176 per 1000	64 fewer per 1000 (124 fewer to 44 more)
Total lymphoceles (Observational studies)	4552 (8 non‐randomised studies)	⨁◯◯◯ Very low^d^ ^,^ ^f^ ^,^ ^g^	OR 0.42 (0.21 to 0.85)	181 per 1000	96 fewer per 1000 (136 fewer to 23 fewer)

^a^ Some concerns for the selection of reported results in the RCTs.

^b^ ROB 2 shows mostly low risk of bias between the included studies.

^c^ Publication bias is strongly suspected.

^d^ Some risk of bias present.

^e^ Very storng association.

^f^ Significant heterogeneity is present.

^g^ Strong association present.

### 2.6 Statistical analysis

Data analysis was conducted using Review Manager (RevMan) version 5.4. Pooled effect estimates were calculated separately for dichotomous and continuous outcomes. For dichotomous variables (e.g., symptomatic lymphoceles, complications and positive margins), results were expressed as odds ratios (ORs) with corresponding 95% confidence intervals (CIs). For continuous outcomes (e.g., operative time, hospital stay, intra‐operative blood loss), mean differences (MDs) and 95% CIs were reported. Heterogeneity across studies was assessed using the Chi‐squared test and displayed using the *I* ^2^ statistic. A value of *I* ^2^ greater than 50% was indicative of substantial heterogeneity. In the presence of substantial heterogeneity, a random‐effects model was applied. Otherwise, a fixed‐effect model was used.

Subgroup analyses were performed to compare outcomes reported by RCTs and observational studies. Funnel plots were constructed to assess the risk of publication bias and small study effects. Statistical significance was defined as a *p*‐value < 0.05 for all analyses.

## 3 RESULTS

**FIGURE 1:**
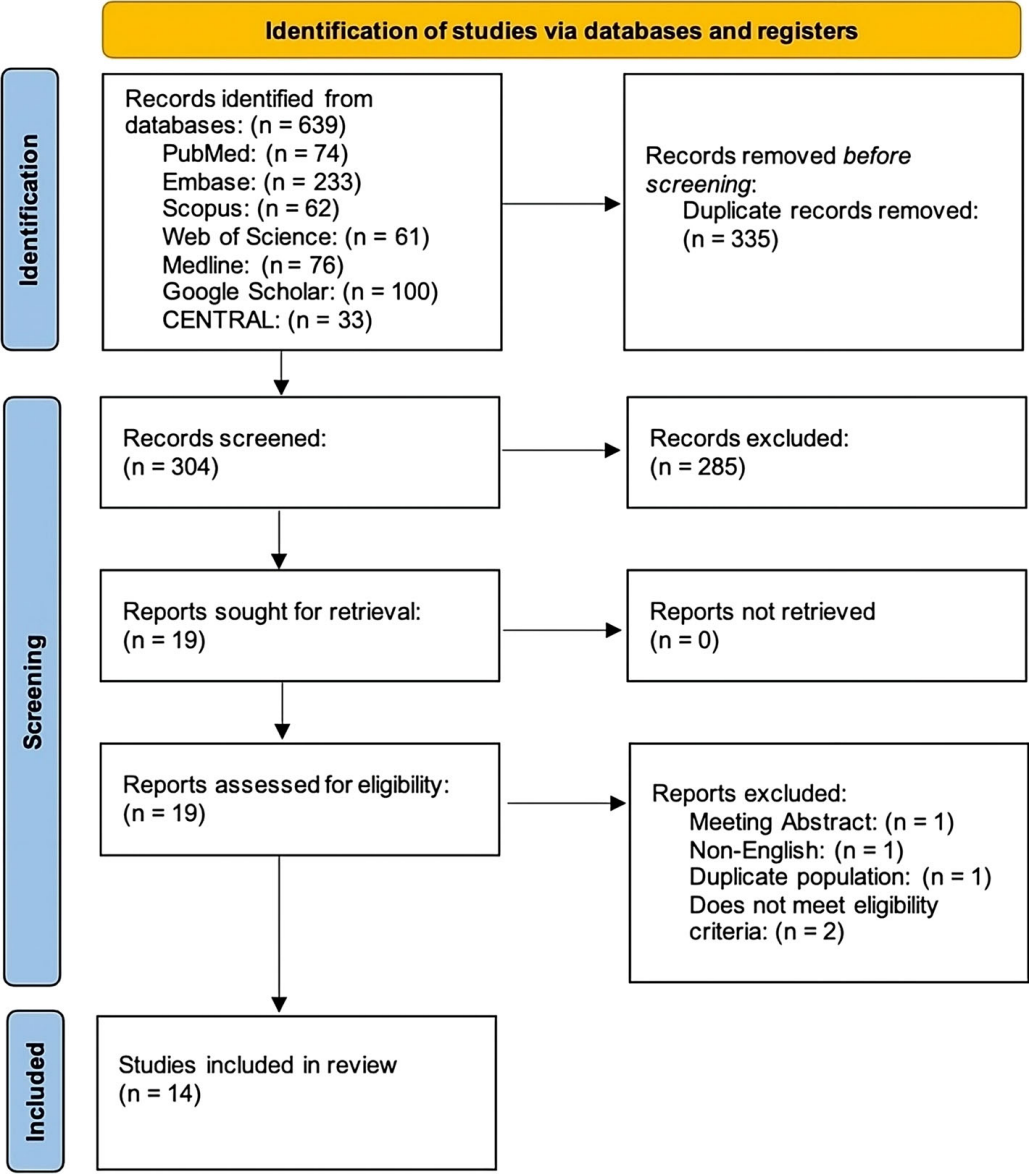
PRISMA flow diagram showing the selection of studies for inclusion and exclusion.

The initial database search identified a total of 639 records. After the removal of 335 duplicates, 304 articles remained for title and abstract screening. Of these, 285 were excluded for not meeting the inclusion criteria. Nineteen full‐text articles were assessed for eligibility. Five were subsequently excluded. One was a meeting abstract, one was not published in English, one was a long‐term follow‐up study with the same population of a study that is already included in the review and two did not meet the predefined eligibility criteria. Fourteen were included in the final review and meta‐analysis. A summary of the selection process is illustrated in the PRISMA flow diagram (Figure 1).

### 3.1 Study characteristics

The 14 included studies comprised six RCTs and eight observational studies. The studies were conducted between 2015 and 2025. There were a total of 7316 patients across the 14 studies. A total of 2997 were in the treatment group and the remaining 4319 were in the control group. The mean age of the participants across the 14 studies ranged from 60 to 68 years old, the mean duration of follow‐up ranged from 0.25 to 24.9 months, and the mean BMI ranged from 25 to 30. A more detailed summary of the study characteristics is provided in Tables 4 and 5.

**TABLE 4:** Study characteristics and mean follow‐up duration.

Study	Year	Study design	Country	PF technique description	Mean follow‐up period (months) ± SD		
					PF	Control	Combined
Pose et al.^14^	2025	RCT	Germany	MT	N/A	N/A	37.1 ± 16.7
Harland et al.^20^	2023	Retrospective	Germany	PPSS	0.25 ± N/A	0.25 ± N/A	0.25 ± N/A
Mathew et al.^21^	2024	Retrospective	India	Peritoneal Distraction	1 ± N/A	1 ± N/A	1 ± N/A
Wagner et al.^18^	2023	RCT	USA	PIF	3.9 ± 1.03	4.2 ± 1.95	N/A
Student Jr et al.^17^	2022	RCT	Czechia	PerFix	19.4 ± 18.4	18.8 ± 18.3	19.1 ± 18.3
Gloger et al.^16^	2022	RCT	Germany	PIF	3 ± N/A	3 ± N/A	3 ± N/A
Lee et al.^22^	2020	Retrospective	USA	PIF	7 ± 2.4	14.3 ± 8.5	N/A
Stolzenburg et al.^23^	2018	Retrospective	Germany	FPPF	3 ± N/A	3 ± N/A	3 ± N/A
Moro et al.^24^	2017	Retrospective	Italy	P.L.E.A.T.	24.9 ± 12.9	63.7 ± 12.6	N/A
Lebeis et al.^8^	2015	Retrospective	USA	PIF	12.5 ± N/A	12.6 ± N/A	N/A
Gamal et al.^25^	2024	Retrospective	USA	PBFB	3 ± N/A	3 ± N/A	3 ± N/A
Neuberger et al.^15^	2024	RCT	Germany	PIF	6 ± N/A	6 ± N/A	6 ± N/A
Gozen et al.^26^	2024	Retrospective	Germany	Peritoneal Flap Fixation with Curling	2 ± N/A	2 ± N/A	2 ± N/A
Brundl et al.^19^	2020	RCT	Germany	PIF	3 ± N/A	3 ± N/A	3 ± N/A

**TABLE 5:** Sample size and baseline characteristics of the included studies.

Study	Sample size (total)			Mean age year ± SD			Mean BMI ± SD		
	PF	Control	Combined	PF	Control	Combined	PF	Control	Combined
Pose et al.^14^	516	536	1052	63.3 ± 6.7	63.7 ± 6.7	63.7 ± 6.7	26.6 ± 3.3	26.7 ± 3.3	26.7 ± 3.3
Harland et al.^20^	91	145	236	65.3 ± 9	65 ± 9	N/A	25.8 ± 3.1	26.35 ± 3.2	N/A
Mathew et al.^21^	180	180	360	65.7 ± 6.1	65.8 ± 6.3	N/A	25.7 ± 2.9	25.1 ± 3.9	N/A
Wagner et al.^18^	110	106	216	N/A	N/A	N/A	N/A	N/A	N/A
Student Jr et al.^17^	123	122	245	63 ± 21.8	64 ± 25.5	64 ± 25.3	30.4 ± 17.7	31.1 ± 17.5	30.7 ± 18.1
Gloger et al.^16^	239	236	475	65 ± 7.5	65.3 ± 7.5	N/A	26.7 ± 2.98	27.3 ± 3.7	N/A
Lee et al.^22^	117	201	318	63.6 ± 6.4	63.4 ± 7.2	N/A	28.6 ± 4.5	29.4 ± 5.0	N/A
Stolzenburg et al.^23^	193	193	386	63.3 ± 22.4	64 ± 25.4	62.3 ± 28.2	30 ± 16	29.6 ± 16.3	N/A
Moro et al.^24^	176	195	371	N/A	N/A	N/A	N/A	N/A	N/A
Lebeis et al.^8^	77	77	154	60.41 ± NA	60.74 ± NA	N/A	28.89 ± NA	29.33 ± NA	N/A
Gamal et al.^25^	567	1700	2267	65 ± 7.4	65 ± 7.4	N/A	28.03 ± 4.1	28.2 ± 4.0	N/A
Neuberger et al.^15^	270	274	544	67.8 ± 6.6	66.2 ± 8.1	67 ± 7.5	27.4 ± 4.5	27.4 ± 3.7	27.5 ± 4.1
Gozen et al.^26^	230	230	460	63.7 ± 25.4	64 ± 24.6	64.7 ± 23.8	29.4 ± 13.1	29.5 ± 12.91	30 ± 22.5
Brundl et al.^19^	108	124	232	64.3 ± 7.1	64.2 ± 4.1	65 ± 7.5	27.6 ± 3.1	27.4 ± 3.7	27.4 ± 3.6

### 3.2 Symptomatic lymphoceles

**FIGURE 2:**
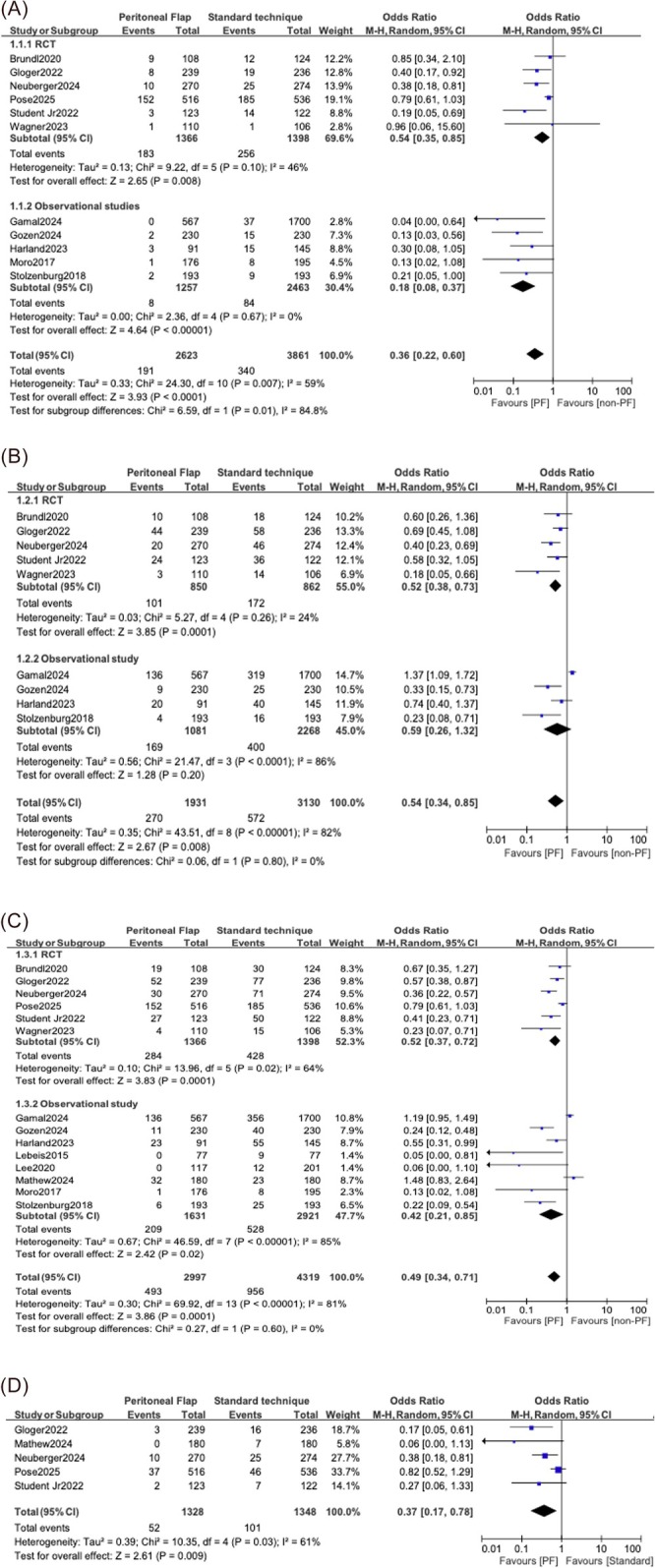
Forest plots comparing the efficacy of the PF against the standard technique of no flap for symptomatic lymphoceles (A), asymptomatic lymphoceles (B), total lymphoceles (C) and lymphoceles needing intervention (D).

Eleven studies involving six RCTs and five observational studies reported on symptomatic lymphoceles. There were 6484 participants between the studies, with 2623 participants in the PF group and 3861 participants in the standard technique group. The RCT subgroup had 2764 participants in total, with 1366 in the PF group and 1398 in the standard technique group. The observational study group had 3720 participants in total, with 2623 in the PF group and 3861 in the standard technique group (Figure 2a).

In the RCT subgroup, the group receiving the PF had fewer events compared to the group receiving the standard technique, producing a pooled OR of 0.54 (95% CI: 0.35–0.85; *p* = 0.008) and moderate heterogeneity (*I* ^2^ = 46%). Whereas the observational subgroup demonstrated a more profound effect (OR: 0.18, 95% CI: 0.08–0.37; *p* < 0.001) with no heterogeneity (*I* ^2^ = 0%). The overall analysis yielded a significant benefit in favour of the PF (OR: 0.36, 95% CI: 0.22–0.60; *p* < 0.001), with moderate heterogeneity (*I* ^2^ = 59%). Significant subgroup differences were observed between the RCTs and observational studies (*p* = 0.01; Figure 2a). Funnel plot suggests a low likelihood of publication bias for the RCTs. However, for observational studies, there is asymmetry around the summary of the estimate of effect, suggesting potential publication bias (Figure S1).

### 3.3 Asymptomatic lymphoceles

Nine studies (five RCTs and four observational studies) encompassing 5061 patients (1931 in the PF group and 3130 in the control group) reported data on asymptomatic lymphoceles. The RCT subgroup involved 1712 participants (850 in the PF group and 862 in the standard technique group), and the observational study subgroup contained 3349 participants (1081 in the PF group and 2268 in the standard technique group; Figure 2b).

Pooled analysis demonstrated that patients undergoing PF formation were significantly less likely to develop asymptomatic lymphoceles compared to those that were managed with the standard surgical approach (OR: 0.54, 95% CI: 0.34–0.85, *p* = 0.008). When studies were stratified by design, the five RCTs demonstrated a statistically significant reduction in asymptomatic lymphocele formation with PF use (OR: 0.52, 95% CI: 0.38–0.73, *p* < 0.001), and low heterogeneity (*I* ^2^ = 24%). In contrast, pooled data from the four observational studies did not demonstrate a statistically significant result (OR: 0.59, 95% CI: 0.26–1.32, *p* = 0.2) and were associated with substantial heterogeneity (*I* ^2^ = 86%). The test for subgroup differences was not statistically significant (*p* = 0.8; Figure 2b). Funnel plot displaying asymmetry around the summary of estimate of effect for both the RCTs and the observational studies suggests that publication bias may be present (Figure S2).

### 3.4 Total lymphoceles

A total of 14 studies (six RCTs and eight observational studies), including 7316 patients (2997 in the peritoneal group and 4319 in the control group), provided data on overall lymphocele formation. The RCT subgroup comprised 2764 patients (1366 in the PF group and 1398 in the standard technique group). The observational studies subgroup encompassed 4552 participants (1631 in the PF group and 2921 in the standard technique group; Figure 2c).

The pooled analysis revealed that the use of a PF significantly reduced the likelihood of total lymphocele formation compared to the standard approach (OR: 0.49, 95% CI: 0.34–0.71, *p* < 0.001). Among the RCT subgroup, PF use was associated with significantly fewer lymphoceles compared to the standard technique group (OR: 0.52, 95% CI: 0.37–0.72, *p* < 0.001), though heterogeneity was moderate (*I* ^2^ = 64%). Similarly, the observational subgroup also favoured PF use (OR: 0.42, 95% CI: 0.21–0.85, *p* = 0.02), though substantial heterogeneity was noted (*I* ^2^ = 85%). The test for subgroup differences between study types was not statistically significant (*p* = 0.6; Figure 2c). Funnel plot shows no publication bias for the RCTs. However, the observational studies are displaying asymmetry. Hence, there is a suspicion of publication bias for the observational studies (Figure S3).

### 3.5 Lymphoceles needing intervention

Five studies, incorporating a total of 2676 participants (1328 in the PF group and 1348 in the standard technique group), reported on the incidence of lymphoceles needing intervention. Pooled analysis demonstrated a significantly lower incidence of intervention‐requiring lymphoceles in the PF group compared to the standard technique group (OR: 0.47, 95% CI: 0.27–0.84, *p* = 0.009). Heterogeneity among the studies was moderate (*I* ^2^ = 61%), indicating some variability in study results. Nonetheless, the overall trend favored the group receiving the PF in reducing the need for intervention (Figure 2d). Funnel plot shows asymmetry suggesting publication bias. However, only five studies are included in the funnel plot, which is not enough to confidently confirm the presence of publication bias (Figure S4).

### 3.6 Total complications

**FIGURE 3:**
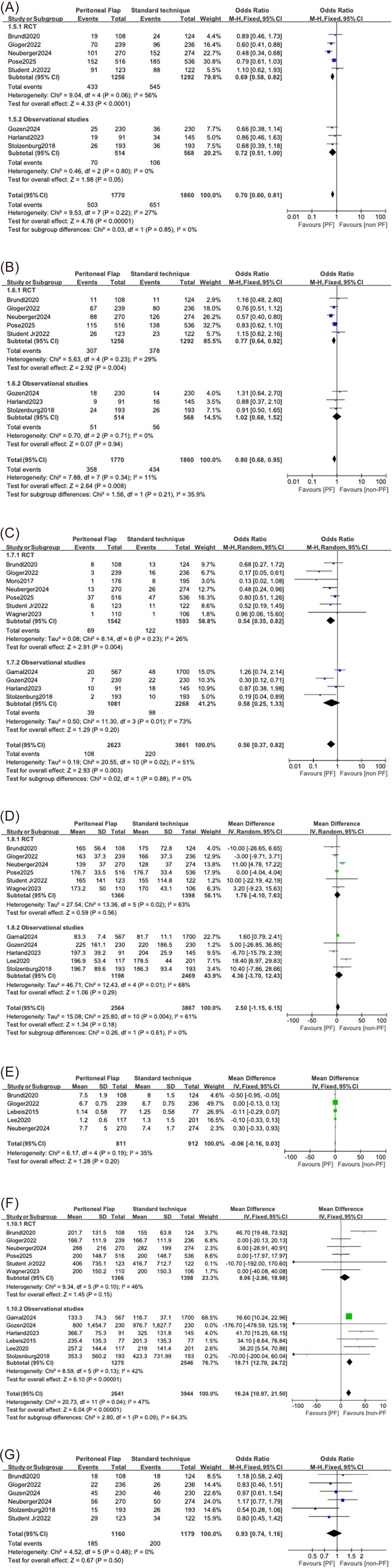
Forest plots displaying the safety of the PF against the standard technique of no flap by comparing total complications (A), minor complications (B), major complications (C), operative time (D), hospitalisation duration (E), intra‐operative blood loss (F) and positive margins (G).

Eight studies, comprising 3630 participants (1770 in the PF group and 1860 in the standard technique group), reported data on total complications defined as total Clavien‐Dindo complications. This included five RCTs involving 2548 participants (1256 in the PF group and 1292 in the standard technique group) and three observational studies involving 1082 participants (514 in the PF group and 568 in the standard technique group; Figure 3a).

When analysed collectively, patients who received the PF experienced significantly fewer complications compared to those treated with the standard approach (OR: 0.69, 95% CI: 0.58–0.82, *p* < 0.001). The RCT subgroup showed a notable reduction in complication rates with the use of a PF (OR: 0.69, 95% CI: 0.58–0.82, *p* < 0.001), with moderate heterogeneity (*I* ^2^ = 56%). Similarly, pooled data from the observational subgroup also showed a borderline statistically significant benefit (OR: 0.66, 95% CI: 0.38–1.14, *p* = 0.05), with no observed heterogeneity (*I* ^2^ = 0%). The test for subgroup differences between the study types was not statistically significant (*p* = 0.85) (Figure 3a). The funnel plot demonstrates no substantial evidence of publication bias (Figure S5).

### 3.7 Minor complications

Eight studies reported on minor complications (Clavien‐Dindo < 3), involving a total of 3630 patients. Of these patients, 1770 received the PF and 1860 received the standard technique. Of the eight studies, five were RCTs and three were observational studies. There were 2548 patients (1256 in the PF group and 1292 in the standard technique group) between the RCTs. Between the three observational studies, there were a total of 1082 participants (514 in the PF group and 568 in the standard technique group; Figure 3b).

Overall, pooled analysis showed that PF use is associated with a statistically significant reduction in minor complications (OR: 0.70, 95% CI: 0.60–0.81, *p* < 0.001). When stratified by study design, the RCT subgroup demonstrated a clear benefit with the use of the PF (OR: 0.69, 95% CI: 0.58–0.82, *p* = 0.004), with low heterogeneity (*I* ^2^ = 29%). Conversely, the observational subgroup did not reveal a statistically significant difference (OR: 0.72, 95% CI: 0.51–1.00, *p* = 0.05), with a heterogeneity of *I* ^2^ = 29%. The test for subgroup differences between study types was not statistically significant (*p* = 0.21; Figure 3b). Funnel plot suggests low risk of publication bias (Figure S6).

### 3.8 Major complications

A total of 11 studies (seven RCTs and four observational studies) encompassing 6484 patients (2623 in the PF group and 3861 in the control group) reported data on major complications (Clavien–Dindo > 2). Within the RCT subgroup, 3135 patients were included (1542 receiving the PF and 1593 receiving the standard technique), while the observational subgroup involved 3349 participants (1081 receiving the PF and 2268 receiving the standard technique; Figure 3c).

Pooled analysis revealed that the PF was associated with a statistically significant reduction in major complications compared to the standard technique (OR: 0.64, 95% CI: 0.47–0.87, *p* = 0.003). When stratified by study design, the RCTs showed a significant benefit favouring PF formation (OR: 0.59, 95% CI: 0.41–0.85, *p* = 0.004), with low heterogeneity (*I* ^2^ = 26%). Conversely, the observational studies did not demonstrate a statistically significant difference (OR: 0.76, 95% CI: 0.50–1.16, *p* = 0.2) and were associated with substantial heterogeneity (*I* ^2^ = 73%). The test for subgroup differences was not statistically significant (*p* = 0.88; Figure 3c). Funnel plot suggests that there is publication bias unlikely (Figure S7).

### 3.9 Operative time

Operative time was reported by 11 studies, involving a total of 6431 patients (2564 in the PF group and 3867 in the standard group). This included six RCTs and five observational studies. In the RCT subgroup, there were a total of 2764 patients (1366 in the PF group and 1398 in the standard technique group). In the observational study subgroup, there were a total of 3667 patients (1198 in the PF group and 2467 in the standard technique group; Figure 3d).

Pooled analysis demonstrated no statistically significant difference in operative time between the PF and standard technique subgroups, with a MD of 2.50 min (95% CI: −1.15 to 6.15, *p* = 0.18). Subgroup analysis of RCTs showed no significant difference (MD: 1.76 min, 95% CI: −4.10 to 7.63, *p* = 0.56; *I* ^2^ = 63%). Similarly, the observational studies did not demonstrate a statistically significant difference (MD: 4.36 min, 95% CI: −3.70 to 12.43, *p* = 0.29; *I* ^2^ = 68%). Heterogeneity across all studies was moderate (*I* ^2^ = 61%), and the test for subgroup differences between study designs was not statistically significant (*p* = 0.61; Figure 3d).

### 3.10 Hospitalisation duration

Five studies encompassing 811 patients in the PF group and 912 patients in the standard technique group reported on hospitalisation duration. The pooled analysis did not demonstrate a statistically significant difference in mean hospital stay between the two groups (MD: −0.06 days, 95% CI: −0.16 to 0.03, *p* = 0.2). Heterogeneity was low (*I* ^2^ = 35%; Figure 3e).

### 3.11 Intra‐operative blood loss

Twelve studies (six RCTs and six observational studies) involving a total of 6585 participants (2641 in the PF group and 3944 in the standard technique group) reported on intra‐operative blood loss. The RCT subgroup comprised 2764 patients (1366 in the PF group and 1398 in the standard technique group), while the observational studies enrolled 3821 patients (1275 in the PF group and 2546 in the standard treatment group; Figure 3f).

Pooled analysis demonstrated that PF use was associated with a statistically significant reduction in intra‐operative blood loss when compared to the standard approach, with a MD of 16.24 mL (95% CI: 10.97–21.50, *p* < 0.001). However, when analyzed by study design, the results differed. Within the RCT subgroup, the MD was not statistically significant (MD: 8.06 mL, 95% CI: −2.86 to 18.98, *p* = 0.15), and there was moderate heterogeneity (*I* ^2^ = 46%). In contrast, the observational studies showed a larger statistically significant reduction in blood loss (MD: 18.71 mL, 95% CI: 12.70 to 24.72, *p* < 0.001), though with a similar degree of heterogeneity (*I* ^2^ = 42%). The test for subgroup differences was not statistically significant (Figure 3f).

### 3.12 Positive margins

Six studies (five RCTs and one observational study) involving a total of 2339 patients (1160 in the PF group and 1179 in the standard technique group) reported on the rate of positive surgical margins. Pooled analysis showed no statistically significant difference between the groups (OR: 0.93, 95% CI: 0.74–1.16, *p* = 0.50), indicating that PF use had no measurable impact on oncological clearance. Heterogeneity among the studies was minimal (*I* ^2^ = 0%; Figure 3g). Funnel plot shows no publication bias (Figure S8).

## 4 DISCUSSION

This systematic review and meta‐analysis investigates the efficacy and safety of the PF during RARP with PLND compared to the standard surgical approach without the PF in preventing the formation of lymphoceles. Pooled analysis of the included studies shows that the PF is associated with a lower incidence of symptomatic lymphoceles, asymptomatic lymphoceles, total lymphoceles and lymphoceles requiring intervention. In terms of safety, total complications, minor complications and major complications are all lower with the PF. Intra‐operative blood loss is lower without the PF. No significant differences were observed in hospital stay and operative time between the two groups.

These results are largely consistent with the results of previously published systematic review literature. Although there are a few differences to note. For example, intra‐operative blood loss is found to be significantly higher with the PF in this review. However, a previous meta‐analysis conducted by De Pinho et al. found that this difference was not statistically significance.^12^ The difference in this finding may be due to our result having lower heterogeneity and greater statistical power. However, it is important to mention that the MD in blood loss in our review was only 16 mL between the two groups. While this result is statistically significant, it is not clinically significant.

Several methods other than the PF have previously been described in preventing the occurrence of lymphoceles. These methods focus on reducing the leakage of lymphatic fluid from lymphatic vessels. Examples include the use of fibrin glue, clips, haemostatic agents and vessel sealing devices.^27^, ^28^, ^29^, ^30^, ^31^ However, none of these methods has produced convincing results.

Stolzenburg et al. created bilateral peritoneal fenestrations during extraperitoneal laparoscopic prostatectomies.^32^ Their results revealed a reduced incidence of both asymptomatic and symptomatic lymphoceles compared to patients who did not receive the fenestrations.^32^ Building on this idea, Lebeis et al. later introduced the PIF.^8^ The PIF was designed to facilitate the flow of lymphatic fluid into the peritoneal cavity. This surgical technique involved suturing of the PF to the lateral border of the bladder, thereby creating a channel in between the PLND bed and the peritoneal cavity.^8^ Lebeis et al. demonstrated impressive results in preventing the formation of symptomatic lymphoceles with the PIF. Since then, many studies have introduced their own modifications to the technique. For example, Moro et al. introduced a modification to the PIF termed ‘preventing lymphocele ensuring absorption transperitoneally’ (P.L.E.A.T.). Their results revealed that P.L.E.A.T. is safe and effective in reducing the formation of lymphoceles. Stolzenberg et al. later followed this with another variation of the PIF termed 4‐point peritoneal fixation (FPPF).^23^ Similarly, they also found a significant reduction in the incidence of lymphoceles in patients who received FPPF. However, shortly after, the first RCT (PIANOFORTE) testing the efficacy of the PIF in reducing postoperative symptomatic lymphocele formation was published^33^. Interestingly, their results opposed those of earlier studies and concluded that the PIF did not prevent the formation of symptomatic or asymptomatic lymphoceles. This was similar to a recent large volume RCT by Pose et al. demonstrating no significant reduction in symptomatic lymphoceles with the use of a PF.^14^ These findings may be a result of considerable variation in the PF technique employed between the included studies. Therefore, efforts must be made to find optimal techniques and to standardise them. Overall, there seems to be a significant reduction in the formation of lymphoceles, including those requiring intervention, with the use of a PF even when considering RCTs alone. Along with this, there is no additional morbidity associated with the PF, making it a safe addition to the standard technique.

The included studies in our systematic review and meta‐analysis have several strengths that enhance the reliability of our findings. A significant proportion of the included studies was prospective RCTs.^14^, ^15^, ^16^, ^17^, ^18^, ^19^ Several studies employed propensity score matching to address confounding factors in retrospective analyses.^21^, ^25^, ^26^ The included studies generally exhibited methodological transparency through detailed descriptions of surgical techniques, giving future studies the information to replicate them.^8^, ^20^, ^24^ However, it is important to acknowledge that this review and its included studies have their limitations. One limitation to note is that many of the included studies varied in the duration of their postoperative follow‐up period. Follow‐up periods varied widely, from as short as 60 days to several months or even years.^16^, ^20^, ^24^, ^26^ Such variations may affect the detection rate of lymphoceles, particularly late‐onset or delayed presentations. Another limitation was due to inconsistent imaging protocols used for detecting lymphoceles across studies. Some studies used ultrasound imaging, while others relied on CT or cystograms.^8^, ^16^, ^17^, ^20^, ^23^ Furthermore, many of the studies were conducted at single institutions, frequently involving only one or two surgeons, which significantly reduces the generalisability of the findings.^18^, ^20^, ^21^, ^22^, ^25^, ^26^ Variations in surgical practice, patient demographics and perioperative management across different centres could influence outcomes considerably. Lastly, although all PF techniques followed the same principle of channelling lymphatic fluid into the peritoneal cavity, there were slight variations in how this was done.^20^, ^22^, ^24^, ^25^ Such variations, although subtle, could have introduced methodological heterogeneity, potentially affecting the pooled estimate of outcomes.

## 5 CONCLUSION

This systematic review and meta‐analysis shows that the PF with RARP and PLND is a safe and effective surgical technique for preventing the formation of lymphoceles. However, it is important to consider that there is notable variation in the PF techniques employed between the studies, and not all the techniques are equally effective. Therefore, efforts must be made to standardise the techniques and to carry out further research, in particular, large‐scale multicentric RCTs.

## AUTHOR CONTRIBUTIONS

**Huseyin Yildiz:** Methodology (equal); data curation (lead); formal analysis (supporting); writing—original draft (lead); visualisation (lead); writing—review and editing (equal); project administration (lead). **Mohammed Zain Ulabedin:** Conceptualization (equal); methodology (equal); data curation (supporting); formal analysis (lead); visualisation (supporting); writing review and editing (equal); supervision (lead); project administration (supporting). **Kevin Byrnes:** Formal analysis (equal); methodology (supporting); data curation (supporting); review and editing (supporting); supervision (supporting). **Benjamin Lamb:** Conceptualization (equal); writing review and editing (equal); supervision (supporting). **David I. Lee:** Conceptualization (equal); writing review and editing (equal); supervision (supporting). **Mohammed Shahait:** Conceptualization (equal); writing review and editing (equal); supervision (supporting).

## CONFLICT OF INTEREST STATEMENT

The author(s) declare(s) no conflict of interest.

## ETHICS STATEMENT

This article is a systematic review and meta‐analysis of previously published studies. No new human or animal participants were involved. Therefore, ethical approval was not required.

## AI DISCLOSURE STATEMENT

The authors did not use generative AI or AI‐assisted technologies in the development of this manuscript.

## Supporting information


**Figure S1.** Funnel plot for symptomatic lymphoceles. Funnel plot assessing publication bias for studies reporting on symptomatic lymphoceles following RARP with PLND.
**Figure S2.** Funnel plot for symptomatic lymphoceles. Funnel plot assessing publication bias for studies reporting on asymptomatic lymphoceles following RARP with PLND.
**Figure S3.** Funnel plot for total lymphoceles. Funnel plot assessing publication bias for studies reporting on total lymphoceles following RARP with PLND.
**Figure S4.** Funnel plot for lymphoceles needing intervention. Funnel plot assessing publication bias for studies reporting on lymphoceles needing intervention following RARP with PLND.
**Figure S5.** Funnel plot for total complications. Funnel plot assessing publication bias for studies reporting on total complications following RARP with PLND.
**Figure S6.** Funnel plot for minor complications. Funnel plot assessing publication bias for studies reporting on minor complications following RARP with PLND.
**Figure S7.** Funnel plot for major complications. Funnel plot assessing publication bias for studies reporting on major complications following RARP with PLND.
**Figure S8.** Funnel plot for positive margins. Funnel plot assessing publication bias for studies reporting on positive margins following RARP with PLND.

**Data S1.** Supporting Information.
