# Supplementary material for: Prevention of lymphoceles using peritoneal flaps during robotic‐assisted radical prostatectomy with pelvic lymph node dissection: A systematic review and meta‐analysis
Source: BJUI Compass. 2026 Feb 26;7(3):e70126. doi: 10.1002/bco2.70126 (PMC12945558; doi:10.1002/bco2.70126)
Supplement: Supplementary file 1 — Figure S1. Funnel plot for symptomatic lymphoceles. Funnel plot assessing publication bias for studies reporting on symptomatic lymphoceles following RARP with PLND. Figure S2. Funnel plot for symptomatic lymphoceles. Funnel plot assessing publication bias for studies reporting on asymptomatic lymphoceles following RARP with PLND. Figure S3. Funnel plot for total lymphoceles. Funnel plot assessing publication bias for studies reporting on total lymphoceles following RARP with PLND. Figure S4. Funnel plot for lymphoceles needing intervention. Funnel plot assessing publication bias for studies reporting on lymphoceles needing intervention following RARP with PLND. Figure S5. Funnel plot for total complications. Funnel plot assessing publication bias for studies reporting on total complications following RARP with PLND. Figure S6. Funnel plot for minor complications. Funnel plot assessing publication bias for studies reporting on minor complications following RARP with PLND. Figure S7. Funnel plot for major complications. Funnel plot assessing publication bias for studies reporting on major complications following RARP with PLND. Figure S8. Funnel plot for positive margins. Funnel plot assessing publication bias for studies reporting on positive margins following RARP with PLND. [file BCO2-7-e70126-s002.docx]

**Supplemental Figures 1 – 8**


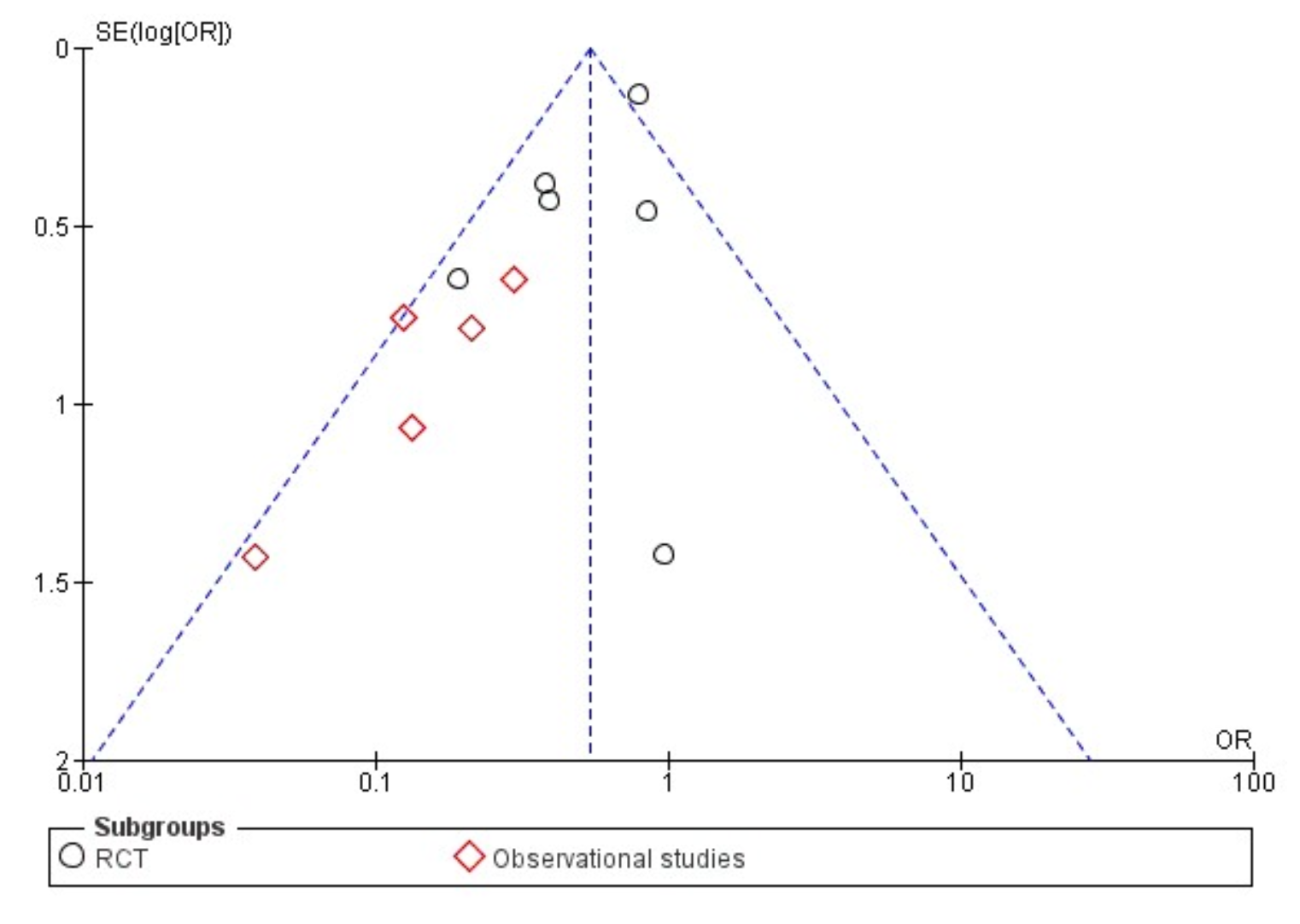


**Figure S1. Funnel plot for symptomatic lymphoceles**. Funnel plot assessing publication bias for studies reporting on symptomatic lymphoceles following RARP with PLND.


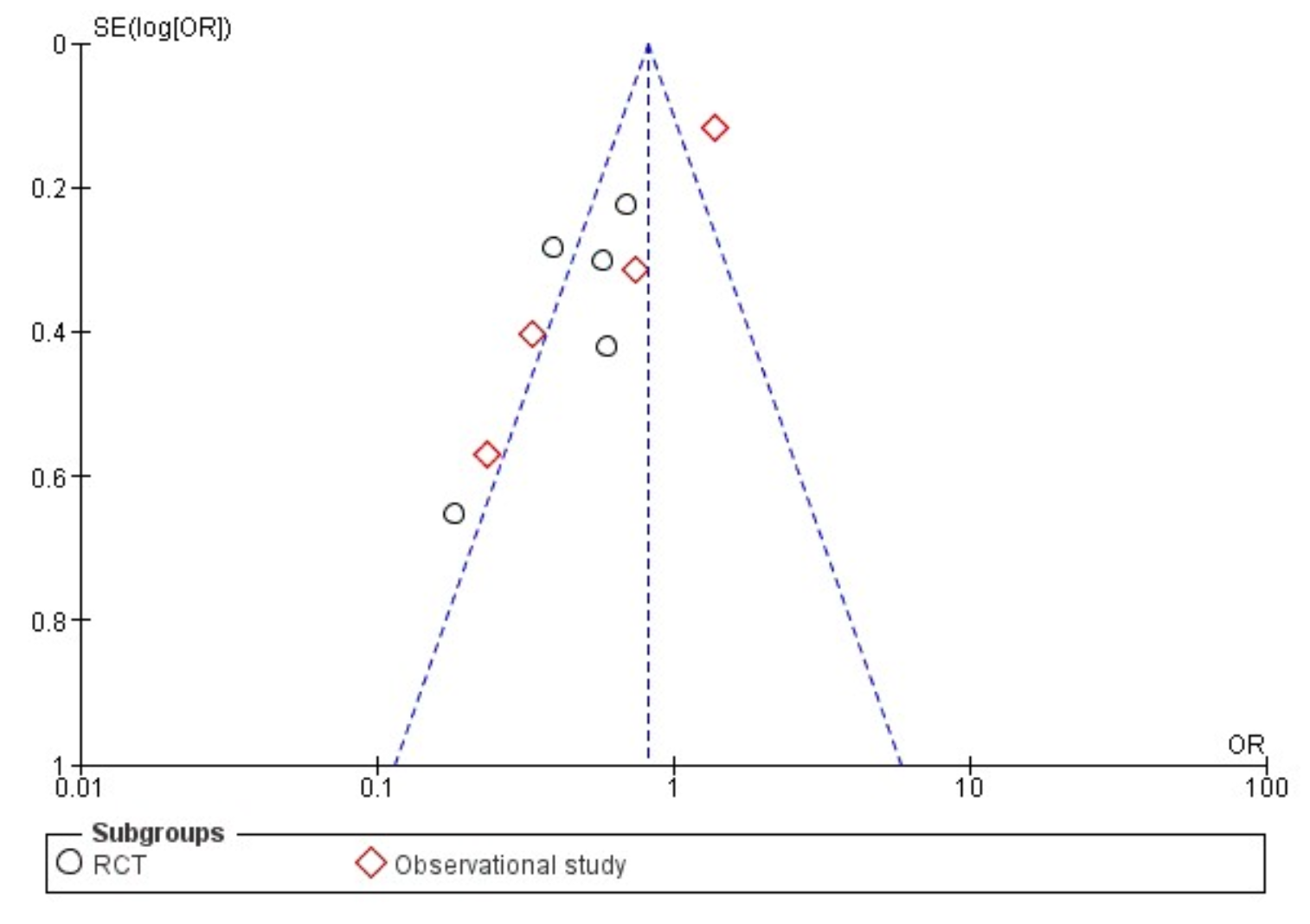


**Figure S2. Funnel plot for symptomatic lymphoceles**. Funnel plot assessing publication bias for studies reporting on asymptomatic lymphoceles following RARP with PLND.


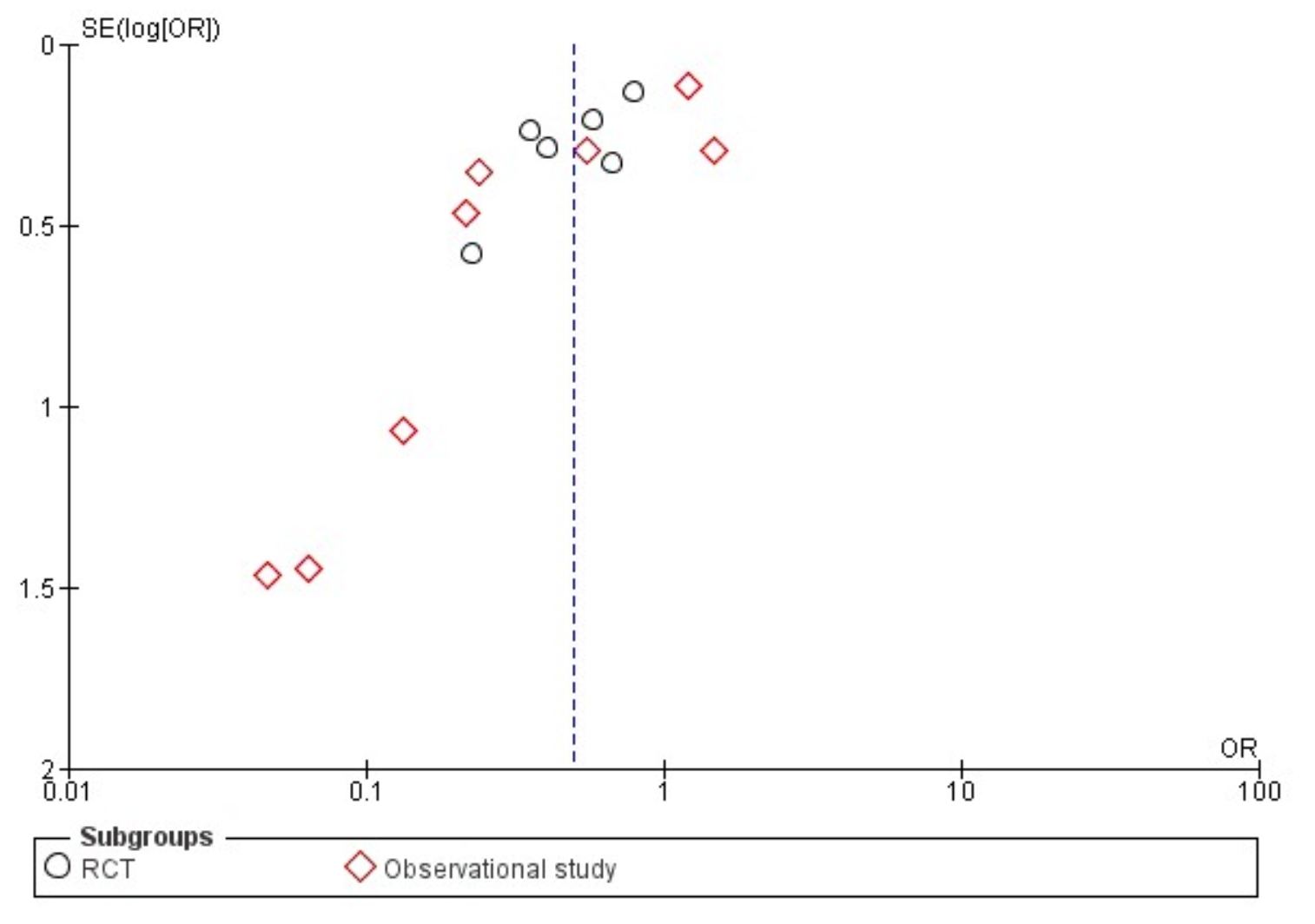


**Figure S3. Funnel plot for total lymphoceles**. Funnel plot assessing publication bias for studies reporting on total lymphoceles following RARP with PLND.


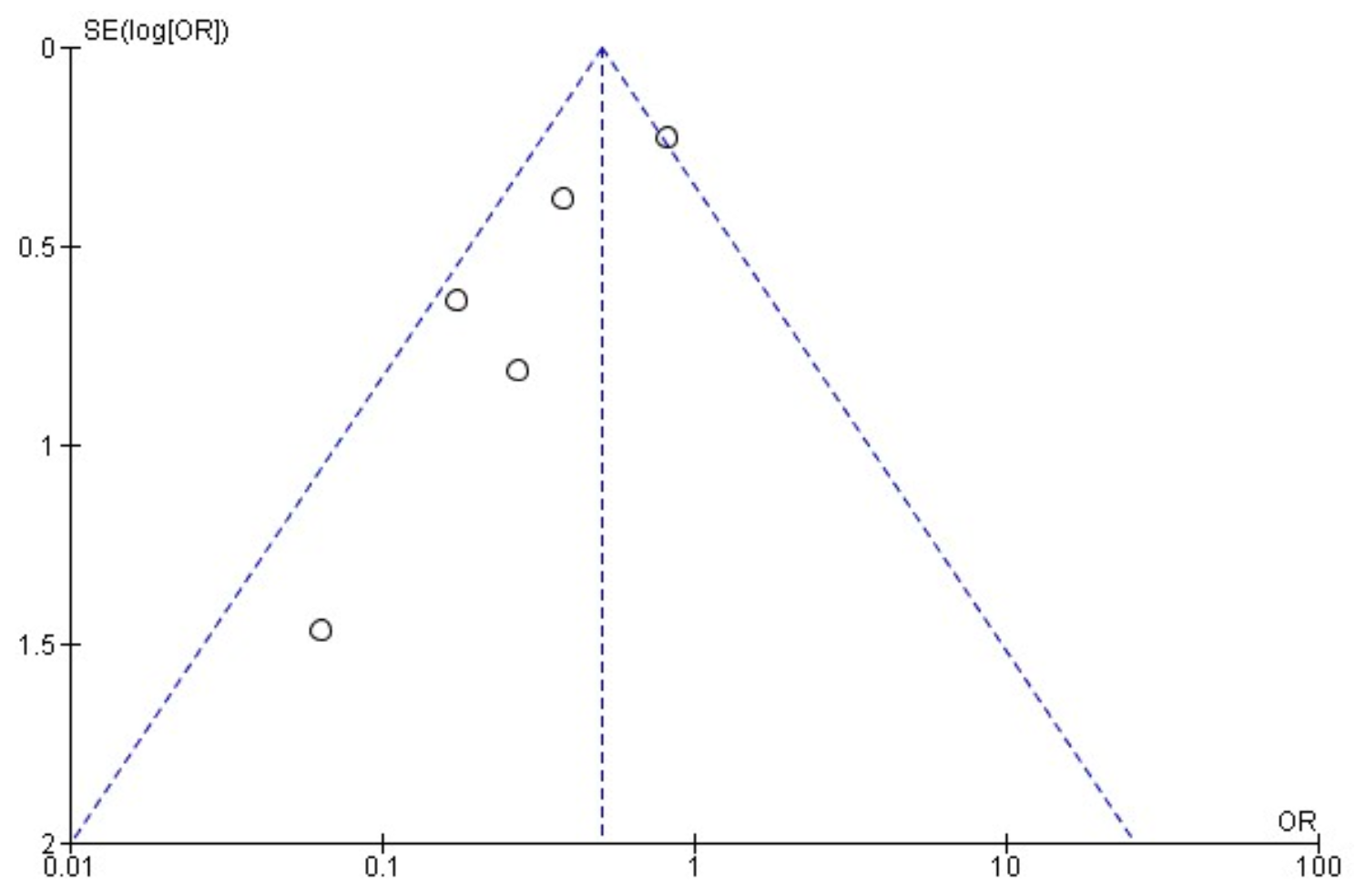


**Figure S4. Funnel plot for lymphoceles needing intervention**. Funnel plot assessing publication bias for studies reporting on lymphoceles needing intervention following RARP with PLND.


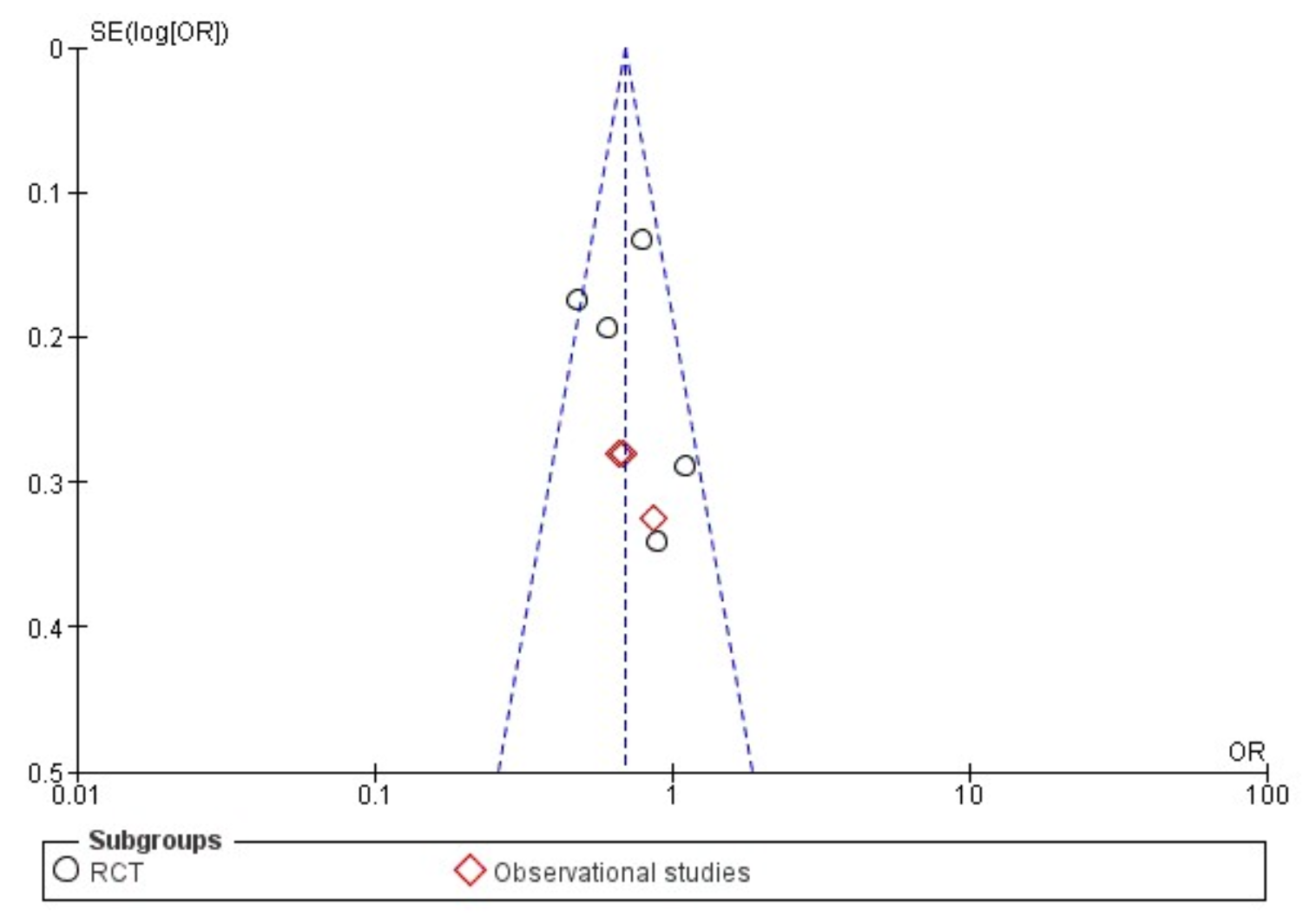


**Figure S5. Funnel plot for total complications**. Funnel plot assessing publication bias for studies reporting on total complications following RARP with PLND.


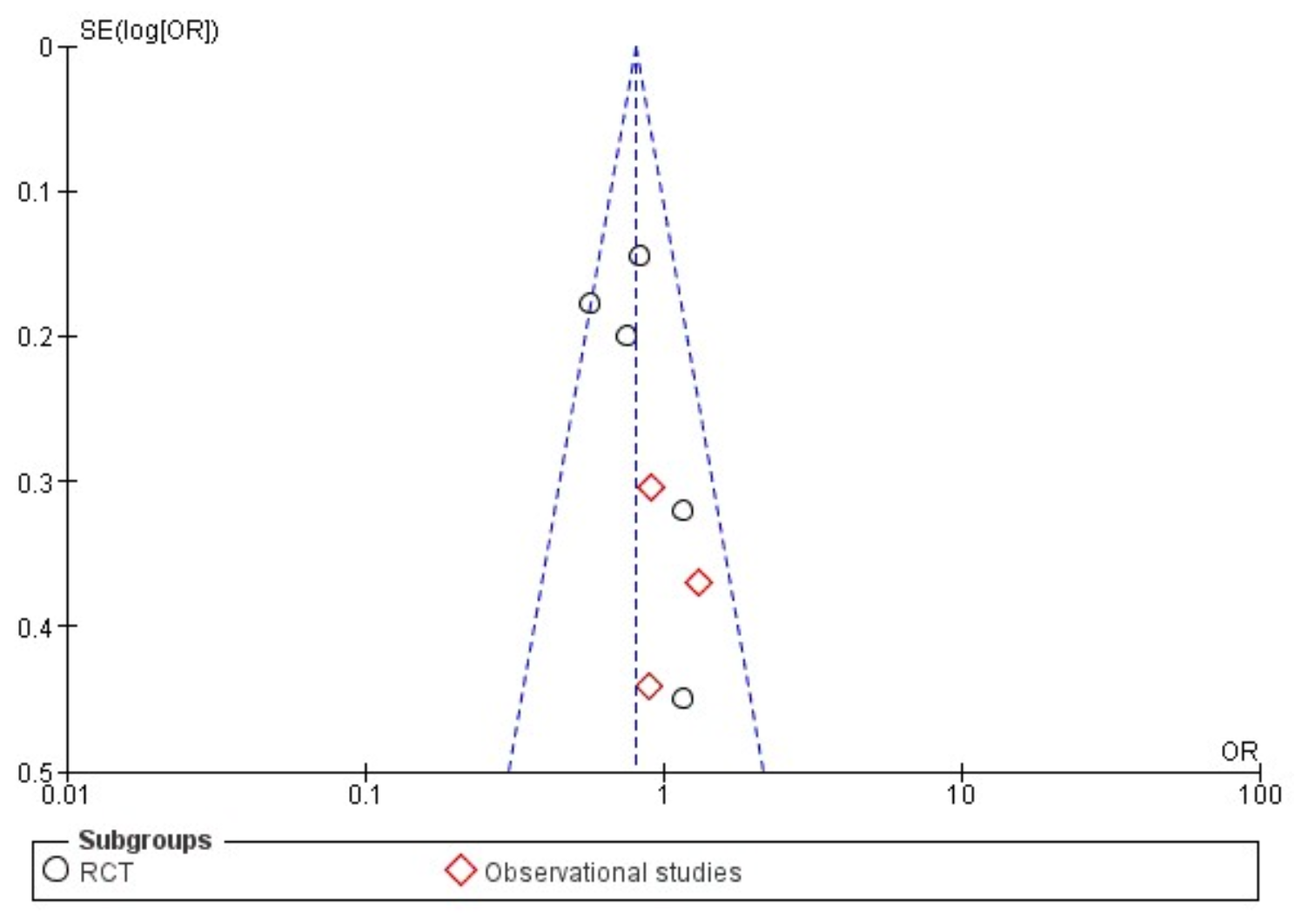


**Figure S6. Funnel plot for minor complications**. Funnel plot assessing publication bias for studies reporting on minor complications following RARP with PLND.


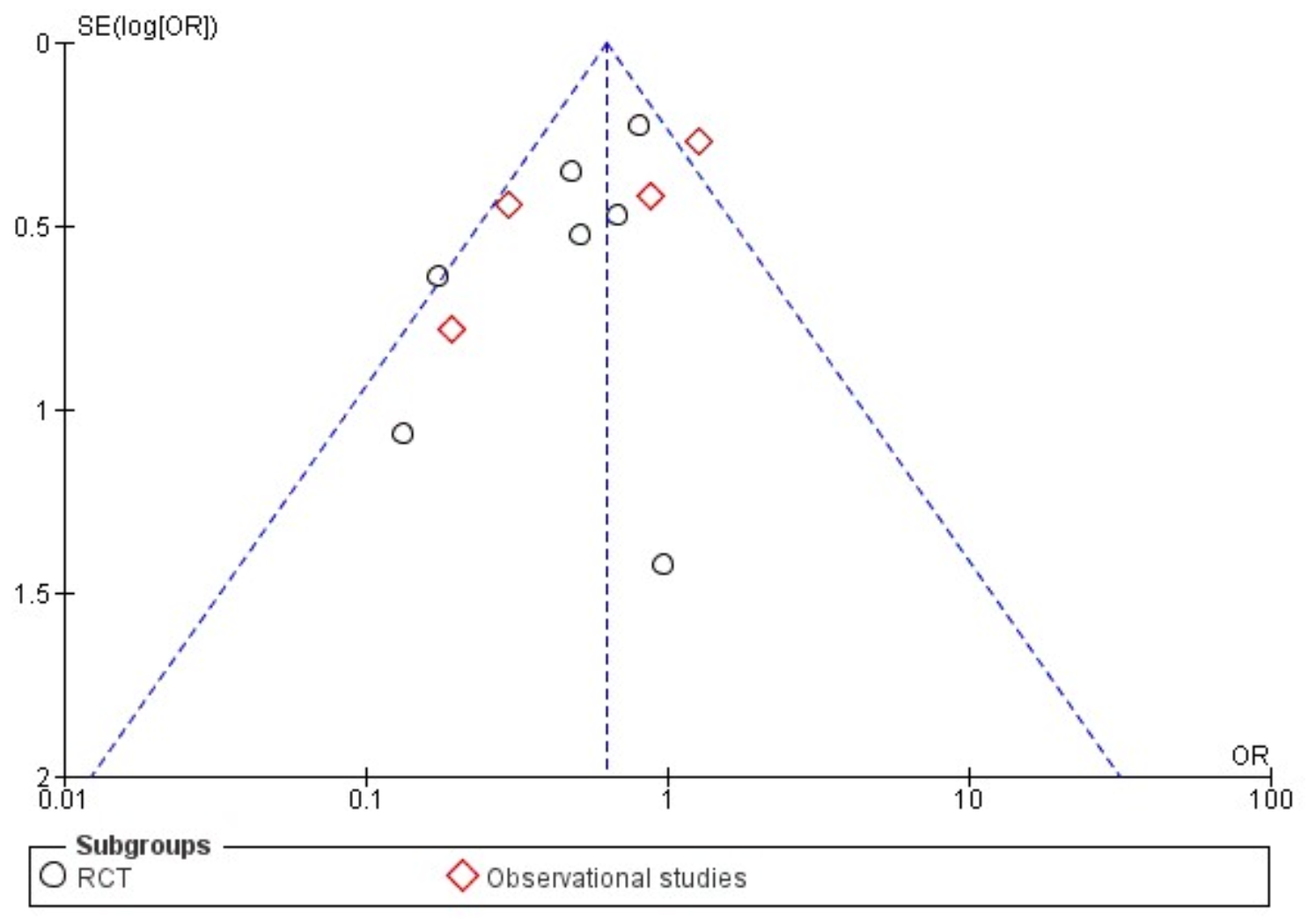


**Figure S7. Funnel plot for major complications**. Funnel plot assessing publication bias for studies reporting on major complications following RARP with PLND.


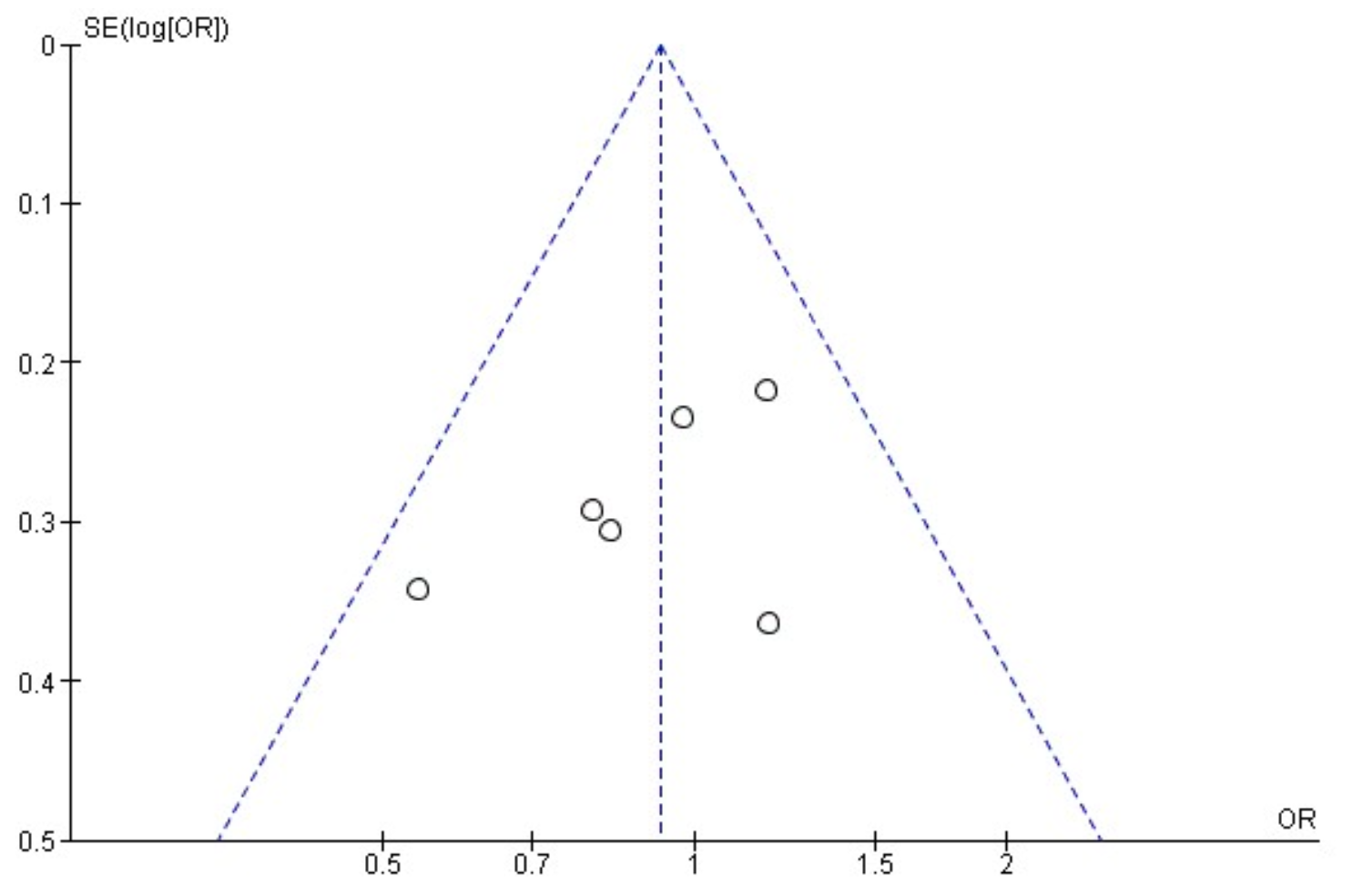


**Figure S8. Funnel plot for positive margins**. Funnel plot assessing publication bias for studies reporting on positive margins following RARP with PLND.
