# Supplementary material for: Prevention of lymphoceles using peritoneal flaps during robotic‐assisted radical prostatectomy with pelvic lymph node dissection: A systematic review and meta‐analysis
Source: BJUI Compass. 2026 Feb 26;7(3):e70126. doi: 10.1002/bco2.70126 (PMC12945558; doi:10.1002/bco2.70126)
Supplement: Supplementary file 2 — Data S1. Supporting Information. [file BCO2-7-e70126-s001.docx]

**Search Terms**

**Concept 1 – Lymphocele**

( (lymphoc*[Title/Abstract] OR symptomatic lymphoc*[Title/Abstract]

**AND**

**Concept 2 – Robotic-assisted prostatectomy**

( "robot-assisted radical prostatectomy"[Title/Abstract] OR "robot assisted radical prostatectomy"[Title/Abstract] OR "robotic-assisted radical prostatectomy"[Title/Abstract] OR prostatectom*[Title/Abstract] OR RARP[Title/Abstract] OR laparo* radical prostat*[Title/Abstract] )

**AND**

**Concept 3 – Peritoneal interposition flap**

( "peritoneal flap"[Title/Abstract] OR "peritoneal flaps"[Title/Abstract] OR "peritoneal interposition"[Title/Abstract] OR "peritoneal fixation"[Title/Abstract] OR peritoneoplasty[Title/Abstract] OR PIF[Title/Abstract] OR peritoneal[Title/Abstract] )

**AND**

**MeSH Term where applicable**

Prostatectomy and Robotic Surgical Procedures and Lymphocele

**SEARCH TERMS FOR EACH DATABASE**

**PubMed:**

(

(lymphoc*[Title/Abstract] OR symptomatic lymphoc*[Title/Abstract])

AND

(

"robot-assisted radical prostatectomy"[Title/Abstract] OR

"robot assisted radical prostatectomy"[Title/Abstract] OR

"robotic-assisted radical prostatectomy"[Title/Abstract] OR

prostatectom*[Title/Abstract] OR

RARP[Title/Abstract] OR

laparo*[Title/Abstract] AND prostat*[Title/Abstract]

)

AND

(

"peritoneal flap"[Title/Abstract] OR

"peritoneal flaps"[Title/Abstract] OR

"peritoneal interposition"[Title/Abstract] OR

"peritoneal fixation"[Title/Abstract] OR

peritoneoplasty[Title/Abstract] OR

PIF[Title/Abstract] OR

peritoneal[Title/Abstract]

)

)

OR

(

"Prostatectomy"[MeSH Terms] AND

"Robotic Surgical Procedures"[MeSH Terms] AND

"Lymphocele"[MeSH Terms]

)

AND english[Filter]

**Embase:**

(

(lymphoc* OR "symptomatic lymphocele"):ti,ab

AND

(

"robot-assisted radical prostatectomy" OR

"robot assisted radical prostatectomy" OR

"robotic-assisted radical prostatectomy" OR

prostatectom* OR

RARP OR

(laparo* AND radical AND prostatectomy)

):ti,ab

AND

(

"peritoneal flap" OR

"peritoneal flaps" OR

"peritoneal interposition" OR

"peritoneal fixation" OR

peritoneoplasty OR

PIF OR

peritoneal

):ti,ab

)

OR

(

'prostatectomy'/exp AND

'robot assisted surgery'/exp AND

'lymphocele'/exp

)

Conference abstracts excluded

Editorials excluded

**Medline:**

(

(lymphoc* OR "symptomatic lymphocele"):ti,ab

AND

(

"robot-assisted radical prostatectomy" OR

"robot assisted radical prostatectomy" OR

"robotic-assisted radical prostatectomy" OR

prostatectom* OR

RARP OR

(laparo* AND radical AND prostatectomy)

):ti,ab

AND

(

"peritoneal flap" OR

"peritoneal flaps" OR

"peritoneal interposition" OR

"peritoneal fixation" OR

peritoneoplasty OR

PIF OR

peritoneal

):ti,ab

)

OR

(

'prostatectomy'/exp AND

'robot assisted surgery'/exp AND

'lymphocele'/exp

)

**Scopus:**

( TITLE-ABS ( lymphoc* OR "symptomatic lymphocele"

)

AND

TITLE-ABS

(

"robot-assisted radical prostatectomy" OR "robot assisted radical prostatectomy" OR "robotic-assisted radical prostatectomy" OR prostatectom* OR rarp OR ( laparo* AND prostat* )

)

AND

TITLE-ABS

(

"peritoneal flap" OR "peritoneal flaps" OR "peritoneal interposition" OR "peritoneal fixation" OR peritoneoplasty OR pif OR peritoneal ) ) OR ( TITLE-ABS ( prostatectomy AND "robotic surgery" AND lymphocele ) )

**CENTRAL:**

(

lymphoc* OR "symptomatic lymphocele" OR lymphocyst*

)

AND

(

"robot-assisted radical prostatectomy" OR

"robot assisted radical prostatectomy" OR

"robotic-assisted radical prostatectomy" OR

prostatectom* OR

RARP OR

(laparo* AND prostat*)

)

AND

(

"peritoneal flap" OR

"peritoneal flaps" OR

"peritoneal interposition" OR

"peritoneal fixation" OR

peritoneoplasty OR

PIF OR

peritoneal

)

OR

(Prostatectomy (mesh) AND Robotic Surgical Procedures (mesh) AND Lymphocele (mesh)

**Web of science:**

(

TS=(lymphoc* OR "symptomatic lymphocele")

AND

TS=(

"robot-assisted radical prostatectomy" OR

"robot assisted radical prostatectomy" OR

"robotic-assisted radical prostatectomy" OR

prostatectom* OR

RARP OR

(laparo* AND prostat*)

)

AND

TS=(

"peritoneal flap" OR

"peritoneal flaps" OR

"peritoneal interposition" OR

"peritoneal fixation" OR

Peritoneoplasty OR

PIF OR

peritoneal

)

**Google Scholar:**

lymphocele|lymphoceles|lymphocyst|"symptomatic lymphocele" "robot-assisted radical prostatectomy"||"robotic-assisted radical prostatectomy"|prostatectomy|RARP "peritoneal flap"|"peritoneal flaps"|"peritoneal interposition"|"peritoneal fixation"|PIF
